# Mesenchymal Stem Cell Therapy Modulates Peripheral–Central Immune Interactions and Attenuates Neuroinflammation-Driven Cognitive Dysfunction

**DOI:** 10.3390/ijms27031182

**Published:** 2026-01-24

**Authors:** Gunel Ayyubova, Shahla Huseynova, Nigar Mustafayeva, Leyla Yildirim, Seher Ismayilova, Tarana Gasimova, Sabina Aliyeva

**Affiliations:** 1 Department of Cytology, Embryology and Histology, Azerbaijan Medical University, Baku AZ1022, Azerbaijan; 2 Department of Human Anatomy and Medical Terminology, Azerbaijan Medical University, Baku AZ1022, Azerbaijan; 3 Genetic Resources Institute of the Azerbaijan National Academy of Sciences, Baku AZ1143, Azerbaijan

**Keywords:** mesenchymal stem cells, neuroinflammation, microglia, cytokines, cognitive dysfunction, lipopolysaccharide

## Abstract

Peripheral inflammation is increasingly recognized as a critical driver of sustained neuroinflammation and cognitive dysfunction in neurodegenerative and inflammation-associated disorders. Systemic inflammatory mediators can compromise blood–brain barrier integrity, activate glial cells, and initiate maladaptive neuroimmune cascades that disrupt hippocampal–prefrontal circuits underlying learning and memory. Here, we investigated whether early systemic administration of human umbilical cord-derived mesenchymal stem cells (hUC-MSCs) mitigates inflammation-driven cognitive deficits in a chronic lipopolysaccharide (LPS) mouse model. Adult mice received daily LPS injections for seven days to induce persistent systemic and central inflammation, which was confirmed by serum and hippocampal cytokine analyses in a separate cohort at the time of MSC administration, followed by intravenous MSC treatment immediately after cessation of the inflammatory insult. Behavioral testing revealed significant impairments in spatial working memory, recognition memory, and associative learning. These deficits were accompanied by pronounced microglial activation, immune cell accumulation, astrocytosis, and a shift toward a pro-inflammatory cytokine milieu with suppression of IL-10 in the hippocampal CA1 region and medial prefrontal cortex. Early MSC treatment attenuated glial reactivity, reduced pro-inflammatory cytokines, restored IL-10 expression, and partially rescued cognitive performance. Collectively, these findings identify a post-inflammatory therapeutic window in which early MSC-based immunomodulation can rebalance neuroimmune signaling and limit inflammation-induced hippocampal–prefrontal circuit dysfunction, highlighting a clinically relevant strategy for targeting cognitive impairment associated with chronic systemic inflammation.

## 1. Introduction

Chronic neuroinflammation is increasingly recognized as a central driver of neural dysfunction across a broad spectrum of conditions, including neurodegenerative diseases, sepsis-associated encephalopathy, and systemic inflammation-induced cognitive impairment. Microglia, the resident immune cells of the central nervous system, are essential for maintaining immune homeostasis but can transition from homeostatic or reparative states toward persistently activated, pro-inflammatory phenotypes under pathological conditions. This shift is accompanied by increased production of cytokines such as IL-1β, TNF-α, and IL-6, activation of inflammasome pathways, oxidative stress, and progressive disruption of synaptic and neuronal integrity [1,2]. Within the hippocampus and connected prefrontal networks—regions critical for learning, memory, and cognitive flexibility—sustained inflammatory signaling perturbs excitatory–inhibitory balance, impairs neurogenesis, and alters synaptic connectivity, ultimately contributing to cognitive dysfunction. Importantly, chronic neuroinflammation reflects a failure of immune resolution rather than a transient defensive response, leading to maladaptive glial activation and persistent circuit dysfunction that underlies long-term cognitive impairment.

Lipopolysaccharide (LPS), a component of the outer membrane of Gram-negative bacteria, is widely used to model systemic inflammation in experimental animals. LPS activates Toll-like receptor 4 (TLR4) on microglia, astrocytes, and endothelial cells, initiating MyD88-dependent signaling cascades that engage NF-κB and MAPK pathways and drive transcription of pro-inflammatory mediators [2]. Prolonged or repeated LPS exposure induces sustained microglial activation, characterized by hypertrophy and increased expression of phagocytic and inflammatory markers such as iNOS, CD68, and complement components C1q and C3, which have been directly implicated in synaptic remodeling and vulnerability [3,4,5]. Importantly, chronic LPS paradigms extend beyond acute sickness behavior and recapitulate features of persistent neuroimmune dysregulation, including long-lasting transcriptomic and epigenetic changes in hippocampal regulatory complexes that may underlie enduring alterations in neuronal and glial function [6,7]. These properties make chronic systemic LPS exposure a relevant model for investigating inflammation-driven cognitive dysfunction and testing immune-modulatory therapeutic strategies.

In parallel, mesenchymal stem cells (MSCs) have attracted considerable interest for their capacity to modulate neuroinflammatory environments. MSCs are multipotent stromal cells that can be isolated from several tissue sources, including bone marrow, adipose tissue, and neonatal umbilical cord, and exhibit low immunogenicity due to limited expression of MHC class II molecules, enabling safe systemic administration [8,9,10]. Rather than acting through direct cell replacement, MSCs exert their therapeutic effects primarily via paracrine mechanisms, including the release of cytokines, growth factors, and extracellular vesicles that suppress pro-inflammatory signaling, limit astroglial reactivity, and promote microglial polarization toward more reparative phenotypes [11,12,13]. Notably, MSC-derived mediators such as IL-10, prostaglandin E_2_, and transforming growth factor-β contribute to immune resolution by inhibiting inflammatory T-cell responses and promoting regulatory immune pathways, while neurotrophic factors, including BDNF and GDNF, support neuronal survival and synaptic stability [9,10,14,15].

Despite substantial preclinical evidence supporting the immunomodulatory and neuroprotective actions of MSCs, their translational relevance in models of sustained systemic inflammation remains incompletely defined. In particular, the impact of treatment timing, the effects on prefrontal cortical pathology, and the relationship between cytokine rebalancing, glial phenotypes, and long-term cognitive outcomes have not been systematically examined in chronic inflammatory paradigms [2,16,17]. Addressing these gaps is critical for understanding whether MSC-based interventions can modify disease trajectories rather than merely suppress acute inflammatory responses.

In the present study, we tested the hypothesis that early post-insult intravenous administration of human umbilical cord-derived MSCs following chronic LPS exposure modulates persistent neuroimmune responses and promotes recovery of cognitive function. Specifically, we investigated whether MSC treatment alters glial activation and cytokine profiles in hippocampal and prefrontal regions and whether these changes are associated with improved performance in memory-dependent behavioral tasks.

## 2. Results

### 2.1. Culture and Identification of Human Umbilical Cord Mesenchymal Stem Cells

**Figure 1:**
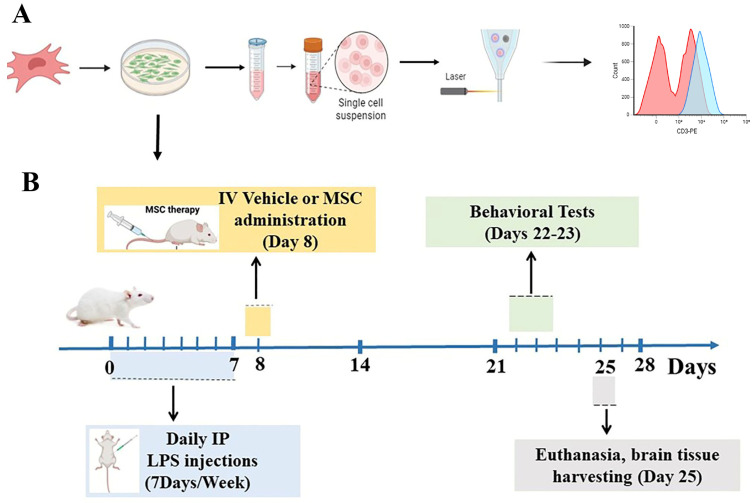
MSC culturing, characterization, and experimental design. (**A**) Schematic representation of the culturing and characterization of human umbilical cord-derived mesenchymal stem cells (MSCs) by flow cytometry. (**B**) Timeline of the in vivo experiment. Mice received daily intraperitoneal LPS injections for 7 days, followed by intravenous vehicle or MSC administration on Day 8. Cognitive tests were conducted on Days 22–23, and brain tissue was collected on Day 25 for immunohistochemical and biochemical analyses.

Isolation, culture, and characterization of human umbilical cord mesenchymal stem cells (hUC-MSCs) were performed with modifications to established protocols [18,19]. Fresh umbilical cords obtained from full-term deliveries were rinsed in sterile phosphate-buffered saline containing antibiotics, and Wharton’s jelly was dissected free of blood vessels. The tissue was minced into ~0.5–1 mm^3^ fragments and subjected to combined enzymatic (collagenase I + hyaluronidase) and mechanical dissociation. The resulting cell suspension was filtered, centrifuged, and resuspended in DMEM/F12 (Gibco, Waltham, MA, USA) supplemented with 10% fetal bovine serum and 1% penicillin/streptomycin. Explants and dissociated cells were cultured at 37 °C in a humidified 5% CO_2_ incubator to allow cell migration and proliferation. Within the first week, elongated, spindle-shaped fibroblast-like cells emerged from tissue fragments and adhered to the flask surface (Figure 1). By days 7–10, extensive outgrowths formed a confluent monolayer, which was passaged at 70–80% confluency using TrypLE Select (Gibco).

**Figure 2:**
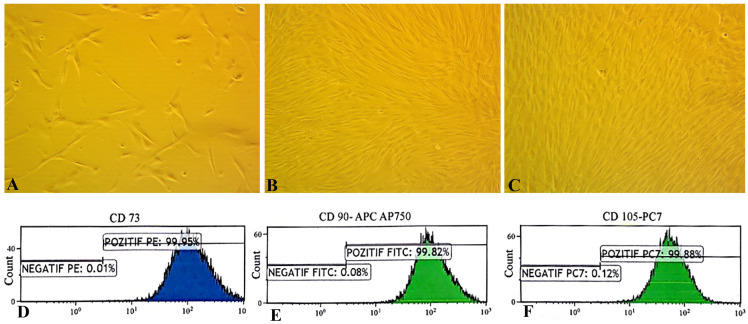
Morphological and immunophenotypic characterization of human umbilical cord mesenchymal stem cells (MSCs). (**A**–**C**) Phase-contrast microscopy of MSCs at passages 1, 2, and 3 showing adherent, spindle-shaped fibroblast-like morphology and increasing cell density (100×). (**D**–**F**) Flow cytometry analysis confirming MSC immunophenotype. Fluorescence-activated cell sorting of hUC-MSCs showed that expression of cell surface markers CD73 (99.95%), CD105 (99.88%), and CD90 (99.82%) was positive. Unstained cells for each condition have been used as the negative controls.

Through successive passages, the cultures became morphologically homogeneous, showing characteristic spindle-shaped cells arranged in swirling, shoal-like clusters (Figure 2A–C). Third-passage hUC-MSCs exhibited vigorous proliferation, following a sigmoidal growth curve with an initial lag phase, a rapid logarithmic expansion, and a plateau phase, reflecting robust self-renewal (Figure 2C). Flow-cytometric immunophenotyping confirmed the canonical MSC profile—positive for CD73, CD90, CD105, CD29, CD44, and CD146 and negative for CD34, CD45, HLA-DR, and CD11b (Figure 2D–F). These findings, together with the surface marker profile and differentiation assays, confirm that the isolated cells were bona fide UC-MSCs with typical morphology, phenotype, and paracrine signatures [19].

We also assessed secreted cytokine profiles of the UC-MSCs. Using multiplex flow cytometry, we quantified interleukins associated with MSC immunomodulation. The flow-cytometric analysis revealed that the UC-MSCs lacked detectable levels of IL-5, IL-6, and IL-8. IL-5 is not typically produced by stromal cells, and IL-6/IL-8 are only secreted when MSCs are primed with inflammatory stimuli. Their absence therefore confirms that the MSCs remained in a quiescent, non-activated state and retained a low-immunogenic, anti-inflammatory phenotype suitable for therapeutic use.

At the time of administration, all UC-MSCs used for transplantation were early-passage cells (P2–P3) with a mean Trypan Blue viability of 94.2 ± 1.9%**,** satisfying the predefined release criterion of ≥90%. The final infusion dose was 1 × 10^6^ viable cells suspended in 100 µL PBS per animal (Figure 1). No cell aggregation or precipitation was observed before injection, and all infusions were completed without complications. Mice exhibited no signs of stress, discomfort, or mortality during or after tail-vein administration, confirming that the UC-MSC preparations were viable, stable, and well-tolerated.

### 2.2. Seven Consecutive Days of LPS Administration Elicited Robust Systemic and Central Inflammatory Responses

Pro-inflammatory cytokine levels, including TNF-α, IL-1β, and IL-6, were quantified in serum and hippocampal lysates obtained 24 h after the final LPS injection. Serum levels of TNF-α (F(5,5) = 24.8, *p* < 0.05), IL-1β (F(5,5) = 2, *p* < 0.05), and IL-6 (F(5,5) = 8.66, *p* < 0.001) were significantly elevated in LPS-treated mice compared with controls, confirming pronounced systemic inflammation. Similarly, hippocampal concentrations of TNF-α (F(5,5) = 4.6, *p* < 0.01), IL-1β (F(5,5) = 3.58, *p* < 0.01), and IL-6 (F(5,5) = 11.57, *p* < 0.05) were significantly increased, indicating sustained neuroinflammatory activation. These findings demonstrate that MSC treatment was initiated during an established inflammatory phase (Supplementary Figure S1).

### 2.3. Behavioral Tests

#### 2.3.1. Open-Field Test (OFT)

**Figure 3:**
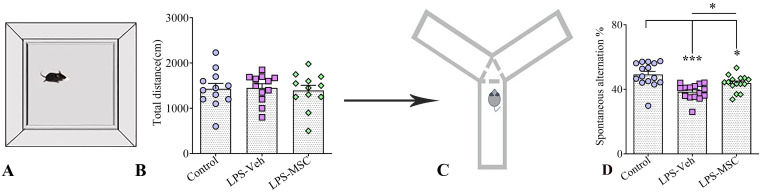
MSC treatment preserves locomotor activity and improves spatial working memory following chronic LPS exposure. (**A**) Schematic illustration of the open-field test and (**C**) Y-maze spontaneous alternation paradigm. (**B**) Total distance travelled in the open-field test did not differ significantly among the experimental groups. (**D**) Spontaneous alternation performance in the Y-maze for control, LPS + vehicle, and LPS + MSC groups. Data are presented as mean ± SEM (*n* = 15 per group). Statistical significance is indicated as * *p* < 0.05 and *** *p* < 0.01.

In the open-field test (OFT), animals from all three experimental groups—naïve controls, LPS-treated mice, and LPS-treated mice receiving MSC therapy—displayed comparable locomotor and exploratory behavior, measured by crossing responses (Figure 3A,B). One-way ANOVA followed by Tukey’s post-hoc tests revealed no significant differences in the total distance travelled among groups, indicating that neither chronic LPS exposure nor MSC treatment altered baseline locomotion. Similarly, the time spent in the center zone did not differ between groups, suggesting that anxiety-like behavior remained unchanged. These findings demonstrate that LPS-exposed mice did not exhibit motor abnormalities during the OFT, and that MSC therapy did not influence general activity. Consequently, any cognitive or behavioral differences observed in other tasks cannot be attributed to alterations in locomotor performance and coordination.

#### 2.3.2. MSCs Improved the Spatial Working Memory in the Model of Neuroinflammation (Y Maze Test)

Spontaneous alternation performance in the Y-maze was used to assess spatial working memory according to established protocols [20] (Figure 3C). Data are presented as mean ± SEM (*n* = 15 per group). Following confirmation of normal data distribution, one-way ANOVA revealed a significant group effect on spontaneous alternation percentage (F(2,42) = 12.22, *p* < 0.0001). Post hoc Tukey’s analysis demonstrated a significant reduction in spontaneous alternation in LPS-Veh mice compared with controls (*p* < 0.001). Importantly, MSC-treated mice showed a significant improvement in spontaneous alternation relative to LPS-Veh animals (*p* < 0.05), although their performance remained modestly lower than that of controls (*p* < 0.05) (Figure 3D).

#### 2.3.3. MSCs Reverse LPS-Induced Impairments in Recognition Memory (Novel Object Recognition Test)

**Figure 4:**
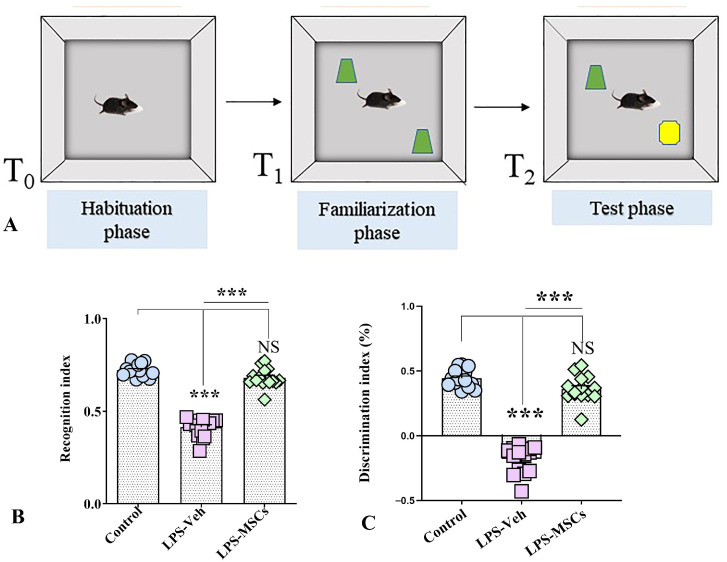
MSC treatment attenuates LPS-induced recognition memory deficits in the Novel Object Recognition test. (**A**) Schematic representation of the Novel Object Recognition (NOR) task showing the habituation phase (T_0_), familiarization phase with two identical objects (shown in green) (T_1_), and the test phase (T_2_), in which one familiar object is replaced by a novel object (shown in yellow). (**B**) Recognition index across experimental groups. (**C**) Discrimination index across experimental groups. Data are presented as individual values with group means. Statistical significance is indicated as *** *p* < 0.001; NS, not significant.

The Novel Object Recognition (NOR) dataset consisted of 15 mice per group (Control, LPS + Vehicle, and LPS + MSC) and demonstrated clear, statistically significant differences in exploratory behavior and recognition memory across groups (Figure 4). During the training phase, all animals explored the two identical objects similarly. However, in the test phase, when one object was replaced with a novel one, LPS-treated mice exhibited a profound deficit in recognition memory, as reflected by markedly reduced preference for the novel object.

Analysis of exploration times revealed that control mice displayed the expected novelty preference, whereas LPS + Vehicle animals explored the novel object for a significantly shorter duration and instead spent a greater proportion of time investigating the familiar object, indicating a loss of novelty recognition. One-way ANOVA confirmed a significant group effect on novel-object exploration (F(2,42) = 55.81, *p* < 0.01), and post-hoc comparisons showed that LPS-treated animals differed significantly from controls, while MSC treatment restored exploration patterns to near-control levels. Correspondingly, the increased familiar-object exploration seen in the LPS + Vehicle group was significantly reduced following MSC treatment (F(2,42) = 43.49; *p* < 0.01), indicating recovery of novelty preference.

To quantify recognition memory more precisely, both the discrimination index (DI) and recognition index (RI) were analyzed (Figure 4B,C). LPS + Vehicle mice showed a severely reduced DI, with values often below zero, reflecting reversed preference and impaired memory. Tukey’s post-hoc test showed that the LPS + Vehicle group had significantly lower DI values compared with both Control and MSC-treated groups (F(2,42) = 198.5; *p* < 0.001), while no difference was found between Controls and MSC-treated mice (*p* > 0.05). These results indicate that MSC treatment rescued the LPS-induced deficit in discrimination ability.

The recognition index (RI) showed an identical pattern. Control animals exhibited high RI values, LPS + Vehicle mice showed a significant reduction, and MSC-treated mice demonstrated values comparable to control animals. ANOVA confirmed a robust group effect, as post-hoc testing showed a highly significant impairment in LPS + Vehicle mice relative to both other groups (F(2,42) = 202.8; *p* < 0.001), with restoration of recognition memory following MSC treatment.

#### 2.3.4. MSC Improved Associative Learning Functions and Long-Term Memory in a Peripherally Induced Model of Neuroinflammation: Passive Avoidance Test (PAT)

**Figure 5:**
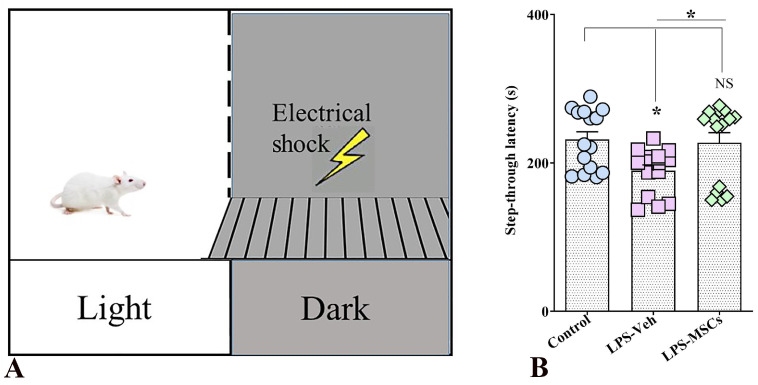
MSC treatment mitigates LPS-induced deficits in aversive memory in the Passive Avoidance test. (**A**) Schematic of the passive avoidance apparatus consisting of a light compartment and a dark compartment in which animals receive a brief foot shock during the training session. (**B**) Bar chart indicates step-through latency during the retention test. Data are presented as individual values with group means. Statistical significance is indicated as * *p* < 0.05; NS, not significant versus control.

Performance in the passive avoidance test was used to assess associative learning and long-term memory retention following chronic peripheral immune activation. Analysis of step-through latency revealed a significant group effect (one-way ANOVA: F(2,42) = 4.55, *p* = 0.016), indicating that LPS exposure and subsequent MSC treatment differentially affected aversive memory performance (Figure 5). Mice exposed to chronic LPS and receiving vehicle displayed markedly reduced latencies to enter the shock-associated dark compartment compared with control animals (*p* < 0.05), reflecting impaired recall of the aversive stimulus and deficient avoidance learning. In contrast, MSC-treated mice exhibited significantly prolonged step-through latencies relative to the LPS + vehicle group (*p* < 0.05), consistent with improved retention of the aversive experience. Notably, step-through latency in MSC-treated animals did not differ significantly from that of control mice (*p* > 0.05), indicating normalization of avoidance memory. Together, these results demonstrate that chronic systemic inflammation disrupts associative learning and long-term memory, whereas early MSC intervention effectively counteracts these deficits and preserves memory retention.

### 2.4. MSCs Prevent LPS-Induced Neuronal Loss

**Figure 6:**
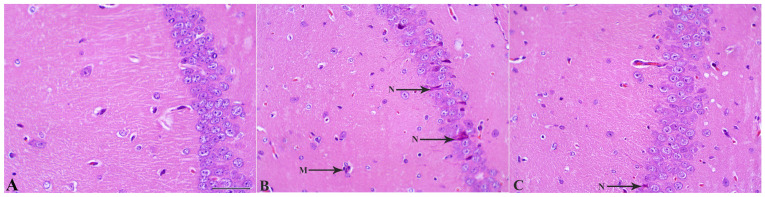
MSC treatment reduces LPS-induced neuronal degeneration in the hippocampal CA1 region. (**A**) Control CA1 tissue showing normal pyramidal neuron morphology with intact cell bodies and organized layering. (**B**) LPS + Vehicle group demonstrating marked neuronal degeneration, including darkly stained, shrunken, and pyknotic neurons (N), along with the clustered three microglial cells (M) in contact with two astrocytes. (**C**) LPS + MSC group showing reduced neuronal damage, with fewer degenerating neurons (arrows) and improved preservation of CA1 structural organization compared with LPS + Vehicle mice. Scale bar = 50 µm.

Investigation of brain tissue sections stained with Hematoxylin–eosin showed that in control mice, the hippocampal architecture is well preserved, with densely packed and orderly arranged pyramidal neurons exhibiting intact morphology and clear nuclear and cytoplasmic boundaries. In contrast, LPS-treated animals display marked structural disorganization within all hippocampal regions, especially CA1. That was seen as a focal atrophy, neuronal rarefaction, and partial disruption of laminar integrity. Approximately 9% of large neurons—most likely pyramidal cells—showed pronounced shrinkage, hyperchromatic cytoplasm, and irregular contours in the LPS + Veh group (Figure 6). Several neurons appeared vacuolated, degenerated, or necrotic, consistent with inflammatory neurodegeneration.

These histological findings demonstrate that MSC administration mitigates LPS-induced neuronal degeneration and preserves hippocampal cytoarchitecture. Building on this evidence of neuroprotection, the next section examines whether MSCs also attenuate microgliosis and modulate the inflammatory cellular milieu underlying neuronal loss.

### 2.5. MSC Treatment Reduces LPS-Induced Microglial Proliferation

**Figure 7:**
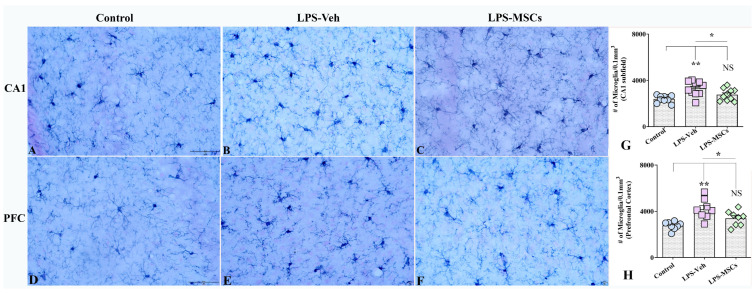
Early administration of MSCs attenuated lipopolysaccharide (LPS)-induced microgliosis in the hippocampal CA1 subfield and prefrontal cortex. Panels (**A**–**C**) illustrate representative examples of IBA1^+^ microglial density in the hippocampal CA1 region from control (**A**), vehicle-treated LPS (LPS + Veh, (**B**)), and MSC-treated LPS (LPS + MSC, (**C**)) groups. Panels (**D**–**F**) show corresponding IBA1^+^ microglial staining in the prefrontal cortex from control (**D**), LPS + Veh (**E**), and LPS + MSC (**F**) animals, demonstrating increased microgliosis following LPS exposure and its attenuation after MSC treatment. Bar charts (**G**,**H**) compare the numbers of IBA1^+^ microglial profiles (per 0.1 mm^3^) in the CA1 region (**G**) and prefrontal cortex (**H**) between experimental groups. Data are presented as mean ± SEM. Scale bar = 50 µm. (* *p* < 0.05; ** *p* < 0.01; NS, not significant).

Stereological quantification of IBA1-positive microglia in different brain regions revealed marked regional differences in response to LPS and MSC treatment (Figure 7). In the CA1 subfield, a one-way ANOVA showed a significant group effect: LPS + Vehicle mice exhibited significantly elevated microglial numbers compared with controls (F = 8.34; *p* < 0.01), indicating robust LPS-induced microgliosis in this region (Figure 7A–C,G). MSC-treated mice showed significantly reduced microglial counts compared to the LPS + Vehicle group (*p* < 0.05), and no significant difference was observed between the MSC-treated and control groups (*p* > 0.05), indicating that MSC therapy effectively normalizes microglial density in CA1. In addition to increased cell numbers, LPS + Vehicle mice displayed distinct perivascular clustering of microglia in CA1, suggestive of altered spatial organization under inflammatory conditions (Supplementary Figure S2A).

A similar pattern was observed in the Prefrontal Cortex (Figure 7D–F,H). Microglial numbers were markedly increased in LPS + Vehicle mice compared with controls (F = 9.3; *p* < 0.01), confirming pronounced microgliosis following LPS exposure. MSC treatment significantly reduced microglial counts in the prefrontal cortex compared with the LPS + Vehicle group (*p* < 0.05), and values did not differ from control animals (*p* > 0.05), indicating a robust increase in microglial numbers in cortical regions involved in higher-order cognitive functions at systemic LPS and effects of MSC treatment. Consistent with hippocampal findings, perivascular microglial clustering was observed in the prefrontal cortex of LPS + Vehicle mice (Supplementary Figure S2B), indicating region-wide alterations in microglial distribution that were attenuated by MSC treatment.

### 2.6. Evaluation of IBA-1 and CD68 Co-Expression in the Hippocampus and Prefrontal Cortex

**Figure 8:**
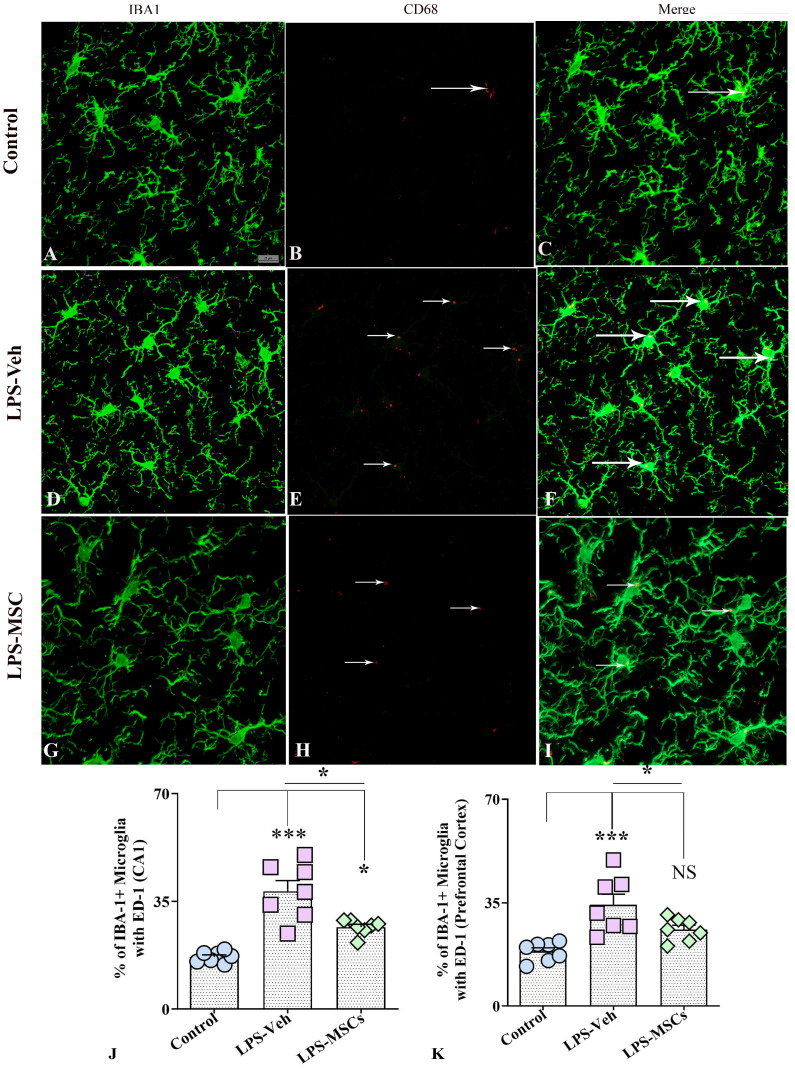
MSCs reduced LPS-induced microglial activation in the hippocampal CA1 region and prefrontal cortex. (**A**–**C**) illustrate representative dual-immunofluorescence images of IBA1^+^ microglia and CD68 (ED1) expression in the hippocampal CA1 region from control (**A**–**C**), vehicle-treated LPS (LPS + Veh, (**D**–**F**)), and MSC-treated LPS (LPS + MSC, (**G**–**I**)) groups. Quantification of IBA1–CD68 co-expression in the hippocampus (**J**) and prefrontal cortex (**K**) demonstrates that chronic LPS exposure markedly increased the proportion of CD68-positive microglia, whereas early MSC treatment reduced microglial activation toward control levels. White arrows indicate the CD68^+^ structures. Data are presented as mean ± SEM. One-way ANOVA with Tukey’s post hoc test was used for statistical analysis (* *p* < 0.05; *** *p* < 0.001; NS, not significant). Scale bar = 25 µm.

Quantitative immunofluorescence analysis of IBA1 and CD68 (ED1) co-expression revealed marked group differences in microglial activation across both the hippocampus and prefrontal cortex (Figure 8). In the hippocampus, a one-way ANOVA demonstrated a highly significant effect of treatment on the proportion of IBA1^+^ microglia expressing the phagocytic marker ED1. LPS + Vehicle mice showed a pronounced increase in IBA1–ED1 co-expression relative to controls (F(2,18) = 25.56; *p* < 0.001), reflecting a strong shift toward a phagocytic, pro-inflammatory microglial phenotype (Figure 8A–G). MSC treatment significantly reduced ED1 co-expression compared with LPS + Vehicle mice (*p* < 0.05), indicating substantial attenuation of LPS-induced microglial activation, although levels remained slightly elevated compared with controls (*p* < 0.05). A similar pattern was observed in the prefrontal cortex (Figure 8H), where a significant treatment effect was also detected. LPS + Vehicle animals again exhibited significantly elevated ED1 co-localization compared with controls (F(2,18) = 11.27; *p* < 0.001), whereas MSC-treated mice showed a robust reduction in ED1 co-expression relative to LPS + Vehicle mice (*p* < 0.05), with values no longer differing from controls (*p* > 0.05).

### 2.7. MSCs Reduce LPS-Induced CD45^+^ Immune Cell Infiltration in the Hippocampus and Prefrontal Cortex

**Figure 9:**
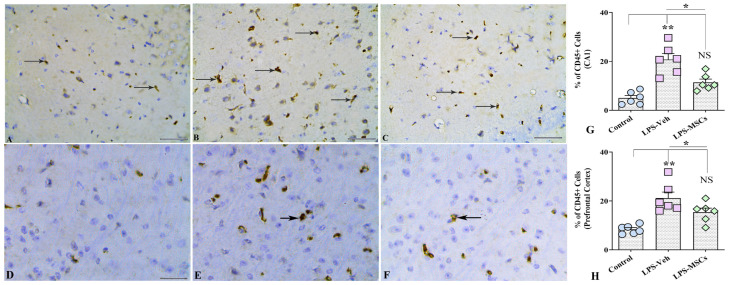
MSCs attenuated LPS-induced CD45^+^ immune cell infiltration in the hippocampus and prefrontal cortex. (**A**–**C**) illustrate representative CD45^+^ immunohistochemical staining in the hippocampal CA1 region from control (**A**), vehicle-treated LPS (LPS + Veh, (**B**)), and MSC-treated LPS (LPS + MSC, (**C**)) groups. Panels (**D**–**F**) show corresponding CD45^+^ staining in the prefrontal cortex from control (**D**), LPS + Veh (**E**), and LPS + MSC (**F**) animals. Bar charts (**G**,**H**) compare the numbers of CD45^+^ cells in the CA1 region (**G**) and prefrontal cortex (**H**) between experimental groups. Black arrows indicate CD45^+^ structures. Data are presented as mean ± SEM. (* *p* < 0.05; ** *p* < 0.01; NS, not significant). Scale bar = 50 µm.

Immunohistochemical analysis of CD45^+^ cells demonstrated a pronounced neuroinflammatory response following chronic LPS exposure, reflected by substantial immune cell infiltration in both the hippocampal CA1 region and the prefrontal cortex (Figure 9A–C,G). In the CA1 subfield, LPS administration induced a striking accumulation of CD45^+^ cells, as confirmed by a highly significant overall treatment effect. Post-hoc comparisons revealed that LPS + Vehicle mice exhibited markedly elevated CD45^+^ cell counts relative to controls (F(2,15) = 21.13; *p* < 0.001). Importantly, MSC treatment produced a robust anti-inflammatory effect, significantly decreasing CD45^+^ cell density compared with the LPS + Vehicle group (*p* = 0.01), effectively restoring cell numbers toward baseline levels observed in untreated controls.

A comparable pattern emerged in the prefrontal cortex. Chronic LPS exposure again resulted in a significant increase in CD45^+^ immune cell infiltration compared with control mice (F(2,18) = 13.08; *p* < 0.01), indicating widespread neuroimmune activation across cortical regions (Figure 9D–F,H). MSC administration significantly mitigated this response (*p* < 0.05), reducing CD45^+^ cell density to levels that were statistically indistinguishable from those of control animals (*p* > 0.05). These results show that suppression of microglial activation through lowering the proportion of cells expressing CD45, as well as CD45^+^ leukocyte infiltration into both hippocampal and cortical structures, MSC therapy helps normalize aberrant neuroimmune activity, contributing to the observed improvements in cognitive outcomes.

### 2.8. MSC Treatment Attenuates LPS-Induced Astrocytic Hyperplasia and Hypertrophy

**Figure 10:**
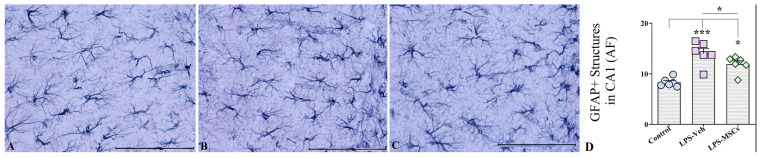
Representative GFAP immunostaining of the hippocampal CA1 region from (**A**) control, (**B**) LPS + Vehicle, and (**C**) LPS + hUC-MSC-treated mice. (**D**) Quantification of GFAP-positive area fraction (AF) in the CA1 region demonstrates increased astrocytic reactivity following LPS administration, which is significantly reduced by hUC-MSC treatment. Data are presented as mean ± SEM. One-way ANOVA with Tukey’s post hoc test; * *p* < 0.05, *** *p* < 0.001. Scale bar = 50 µm.

Next, we performed the quantitative analysis of GFAP^+^ astrocytes in the hippocampus CA1 area and observed marked alterations in astroglial morphology and density across the treatment groups. J image analyses of areas occupied by GFAP positive structures with following statistical evaluation of obtained results using a one-way ANOVA demonstrated a highly significant treatment effect on GFAP^+^ cell numbers (Figure 10). LPS + Vehicle mice exhibited a robust increase in GFAP^+^ astrocyte density relative to controls (F(2,15) = 16.92; *p* < 0.001), reflecting both astrocytic hyperplasia (increased cell numbers) and hypertrophy (enlarged soma and thickened processes)—hallmarks of reactive astrogliosis (Figure 10A–C). MSC treatment markedly mitigated this astroglial response. Mice receiving intravenous MSCs displayed significantly fewer GFAP^+^ cells than their LPS + Vehicle counterparts (*p* < 0.05), indicating that MSCs effectively dampened LPS-induced astrocytic activation (Figure 10D). Although GFAP^+^ cell counts in MSC-treated animals remained moderately elevated compared with controls (*p* < 0.05), the reduction relative to LPS-challenged mice was substantial, demonstrating partial but biologically meaningful normalization of astrocyte reactivity. Beyond quantitative reductions, MSC therapy also qualitatively reduced the severity of astrocytic hypertrophy, with GFAP^+^ cells exhibiting smaller somata and more refined processes compared with the pronounced morphological activation observed in the LPS + Vehicle group. These findings suggest that MSCs not only suppress astrocytic proliferation but also modulate morphological remodeling associated with neuroinflammation.

### 2.9. Biochemical Analysis of Hippocampal Cytokine Levels

**Figure 11:**
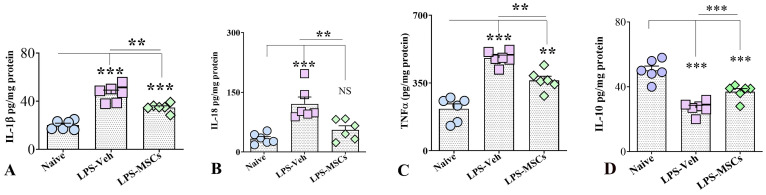
hUC-MSC treatment normalizes hippocampal cytokine levels following chronic LPS exposure. Levels of (**A**) IL-1β, (**B**) IL-18, (**C**) TNF-α, and (**D**) IL-10 in hippocampal tissue from naïve control, LPS + Vehicle, and LPS + hUC-MSC–treated mice. Chronic LPS increased pro-inflammatory cytokines and reduced IL-10, whereas hUC-MSC treatment attenuated inflammatory signaling and restored IL-10 levels. Data are presented as mean ± SEM. One-way ANOVA and Tukey’s post hoc test were used. ** *p* < 0.01; *** *p* < 0.001. NS, not significant.

Because hippocampal function is critically involved in spatial, recognition, and associative memory performance, cytokine measurements were focused on hippocampal tissue to provide a region-specific molecular assessment of inflammation relevant to the behavioral outcomes. We quantified the levels of key pro-inflammatory and anti-inflammatory cytokines in hippocampal tissue lysates taken from all three animal groups. ELISA measurements showed that chronic LPS administration triggered a robust inflammatory response across all examined markers (Figure 11). IL-1β (F(2,15) = 40.94; *p* < 0.001) and TNF-α were markedly elevated in LPS-Veh mice (F(2,15) = 39.38; *p* < 0.001), and although MSC treatment significantly reduced their levels (*p* < 0.01), cytokine concentrations remained higher than in controls, indicating partial normalization. In contrast, IL-18 exhibited a clearer therapeutic response: LPS robustly increased IL-18 (F(2,15) = 14.29; *p* < 0.001), but MSC administration reduced expression to values indistinguishable from controls (*p* > 0.05).

In contrast, evaluation of the anti-inflammatory cytokine IL-10 revealed a marked suppression following LPS exposure. IL-10 levels in brain homogenates were significantly reduced in vehicle-treated LPS mice compared with controls, whereas early MSC administration significantly increased IL-10 expression (Figure 11). One-way ANOVA and post hoc analysis demonstrated that IL-10 was markedly reduced in LPS-Veh animals relative to controls (F(2,5) = 30.81; *p* < 0.001), while MSC treatment significantly restored IL-10 levels compared with LPS-Veh mice (*p* < 0.001), although values remained below control levels (*p* < 0.001).

Thus, although not all cytokine concentrations returned completely to control levels, the overall pattern strongly supports a broad anti-inflammatory action of MSCs, consistent with the structural and behavioral improvements observed in treated animals.

## 3. Discussion

Chronic systemic LPS administration in our model induced a clear and persistent neuroinflammatory state, reflected by microglial activation, astrocytosis, elevated cytokine levels, and marked impairments in multiple forms of memory. These features are consistent with well-established models of inflammation-driven neurodegeneration, where LPS activates the TLR4/MyD88/NF-κB signaling cascade, provokes oxidative stress, and leads to persistent alterations in hippocampal function [16,21]. Importantly, early intravenous administration of MSCs—initiated immediately after completion of the inflammatory insult—substantially attenuated these pathological changes. MSC-treated animals exhibited reduced glial reactivity and cytokine burden, accompanied by significant recovery of spatial working memory, recognition memory, and associative learning.

Longitudinal and population-based studies demonstrate that elevated systemic inflammatory markers—particularly IL-6, TNF-α, IL-1β, and CRP—are associated with accelerated cognitive decline and increased conversion from mild cognitive impairment to AD [22,23]. Large-scale biomarker analyses of recent reports further demonstrate that systemic inflammatory signatures predict hippocampal atrophy and amyloid/tau pathology years before the emergence of clinical symptoms, supporting a causal relationship between chronic peripheral inflammation and AD-related neurodegeneration [24,25]. Similar chronic cognitive problems are seen in survivors of sepsis and systemic infections, who frequently develop persistent neuroinflammation and disrupted hippocampal–prefrontal circuits [26,27]. Mechanistic investigations have shown that elevated systemic IL-1β, TNF-α, IL-18, and alarmins, such as HMGB1, weaken BBB integrity, prime microglia, and activate complement and inflammasome pathways [28,29], providing a strong clinical and mechanistic framework for the chronic LPS model used in the present study.

A key feature of the present study is the timing of MSC administration. In contrast to paradigms targeting either acute inflammation or advanced neurodegeneration, MSCs were administered intravenously on day 8, immediately after completion of the 7-day LPS regimen and at a stage when systemic and central inflammation were evident, as confirmed by cytokine profiling. This time point corresponds to an early post-insult phase in which neuroinflammatory processes have been initiated but are not yet fully stabilized. Previous studies indicate that neuroinflammatory markers, including TNF-α, IL-1β, and Iba1, as well as alterations at the blood–brain barrier (BBB), remain detectable for at least 7–10 days following systemic LPS exposure, suggesting the presence of a therapeutically relevant window [5]. Additionally, consistent with prior reports demonstrating MSC activity in inflamed brain regions following systemic administration [11,30,31,32], the timing strategy used in our study reflects clinically relevant conditions in which inflammation is established but remains modifiable.

Recent studies on MSC-based drug delivery indicate that systemically administered MSCs can interact with the neurovascular unit under inflammatory conditions [30]. MSCs have been shown to serve as effective carriers for oncolytic viruses in glioma, as umbilical cord–derived MSCs loaded with IL-24– and/or endostatin-expressing adenoviruses or CRAd.S.pK7 enhanced tumor-specific apoptosis, inhibited angiogenesis, and prolonged survival [30,31]. Reports indicate that MSCs express chemokine and adhesion receptors, such as CXCR4 and VLA-4, which enable them to respond to inflammatory cues released by activated endothelium, astrocytes, and microglia, thereby enhancing their migration to brain tissue, particularly when the blood–brain barrier is compromised [30].

In accordance with previous studies, chronic LPS exposure disrupts multiple cognitive domains, including spatial working memory, recognition memory, and associative learning, reflecting dysfunction within hippocampal CA1–subiculum and medial prefrontal circuits that are highly vulnerable to inflammatory injury [32,33]. Early intravenous MSC administration markedly improved performance across all behavioral paradigms, indicating broad restoration of these inflammation-sensitive neural networks. These findings are strongly supported by multiple independent studies demonstrating that intravenously delivered MSCs can rescue cognitive deficits in LPS-induced neuroinflammation, AD pathology, and ischemic brain injury, even when administered after the inflammatory insult [10,32,34,35,36,37,38,39,40]. Wharton’s Jelly-derived MSCs improved spatial learning and attenuated memory deficits in APP/PS1 transgenic mice following intravenous administration, effects that had been associated with increased IL-10 expression, reduced microglial activation, and decreased pro-inflammatory cytokines such as IL-1β and TNF-α [34]. Moreover, the translational relevance of our findings is further supported by evidence from large-animal models of multiple sclerosis [35]. In dogs with naturally occurring demyelinating leukoencephalitis—a spontaneous, immune-mediated CNS disorder closely resembling multiple sclerosis—systemic MSC administration stabilized neurological function and attenuated disease progression. Notably, MSC treatment in this model is reported to act primarily through suppression of ongoing neuroinflammation rather than direct tissue replacement, consistent with a disease-modifying mechanism [35]. The demonstrated efficacy and safety of systemic MSC delivery in this chronic MS-like condition further strongly support the clinical feasibility of intravenous MSC therapy.

Consistent with the observed behavioral recovery, histological analyses demonstrated that chronic LPS exposure induced pronounced microgliosis, CD45^+^ immune cell infiltration, CD68^+^ phagocytic activation, and astrocytosis in the hippocampal CA1 region and prefrontal cortex, whereas early MSC treatment significantly attenuated these neuroimmune alterations. Similar reductions in microglial activation and immune cell recruitment following intravenous MSC administration have been reported across models of systemic inflammation, traumatic brain injury, and neurodegeneration, underscoring the robustness of this immunomodulatory effect [11,36]. Notably, Park et al. (2015) demonstrated that MSCs regulate astrocytic end-foot organization, restore tight junction protein expression, and suppress inflammatory astrocyte reactivity, thereby preserving blood–brain barrier (BBB) integrity and limiting peripheral immune cell infiltration into the brain parenchyma [37]. This BBB-stabilizing action provides a plausible explanation for the reduced CD45^+^ immune cell accumulation and attenuated astrocytosis observed in MSC-treated animals [38,39,40]. The perivascular clustering of microglia observed in both hippocampal and prefrontal regions under chronic LPS exposure further supports the involvement of neurovascular-associated microglial responses in sustained neuroinflammation and is consistent with the attenuation of these spatial alterations following MSC treatment.

At the mechanistic level, chronic TLR4-driven inflammation promotes a reactive, phagocytic microglial phenotype characteristic of disease-associated microglia (DAM), marked by increased CD68 expression and accompanied by astrocytic activation—features resembling early neurodegenerative pathology [39,40]. Furthermore, microglia upregulate CD45, reflecting a primed inflammatory state with enhanced phagocytic capacity. Taking into consideration that CD45 immunohistochemistry used in this study does not allow discrimination between CD45^low resident microglia and CD45^high infiltrating leukocytes, CD45^+^ cells should be interpreted as representing an activated myeloid population rather than a specific cell lineage. Importantly, recent evidence highlights that CD45 expression in the inflamed or aged brain reflects microglial activation state rather than strict cellular origin, particularly in perivascular and neurovascular niches where resident microglia and peripheral myeloid cells exhibit overlapping marker profiles [41]. Previous reports have shown that microglia can upregulate CD45 under conditions of CNS injury and chronic inflammation. Within this interpretative framework, systemic LPS exposure is considered to promote a shift from homeostatic toward primed and disease-associated microglial-like activation states in chronically inflamed CNS tissue [42].

In parallel with these cellular changes, cytokine analyses provided molecular evidence of sustained neuroimmune priming. Chronic LPS exposure increased pro-inflammatory cytokines (IL-1β, TNF-α, IL-18) while suppressing the anti-inflammatory cytokine IL-10, reflecting impaired inflammatory resolution that favors prolonged microglial activation. Early intravenous MSC administration reversed this cytokine imbalance, restoring IL-10 and attenuating inflammatory signaling, which was accompanied by a significant reduction in CD45^+^/CD68^+^ reactive myeloid cells in both the hippocampus and prefrontal cortex. Given the context-dependent regulation of CD45 and CD68 expression during neuroinflammation, this reduction most likely reflects attenuation of neuroimmune activation and phagocytic reactivity, rather than selective depletion of a single myeloid population [40]. This shift toward a more homeostatic neuroimmune profile is consistent with the established immunomodulatory and neuroprotective properties of MSCs [9,43,44]. Notably, the reduction in CD68^+^ phagocytic microglia aligns with evidence that systemically delivered MSCs suppress pro-inflammatory microglial signaling and promote a transition toward reparative, M2-like phenotypes, thereby preserving synaptic integrity and supporting cognitive recovery [36,43].

Collectively, these data support a model in which systemically administered hUC-MSCs exert therapeutic effects on the inflamed brain primarily through paracrine immunomodulation and neurovascular interactions, rather than direct parenchymal engraftment; notably, direct tracking of MSC biodistribution and localization was beyond the scope of the present study and should be addressed in future investigations. Consistent with previous reports, MSCs release soluble immunomodulatory mediators such as IL-10, TGF-β, and prostaglandins that suppress NF-κB- and p38 MAPK-dependent inflammatory signaling in peripheral immune cells and brain-resident glia [12,32,38,44].

In parallel, MSC-derived trophic factors, including BDNF, NGF, VEGF, and Ang-1, as well as extracellular vesicles carrying regulatory microRNAs, support neuronal survival, synaptic stability, angiogenesis, and vascular integrity. Although limited MSC migration to the CNS may occur under conditions of blood–brain barrier dysfunction [30], extensive parenchymal engraftment is not required; by sensing inflammatory chemokine gradients and interacting with the neurovascular interface, MSCs can preferentially deliver these immunoregulatory and neuroprotective signals to inflamed CNS regions, thereby reshaping the neuroinflammatory milieu and promoting functional recovery in chronic neuroinflammatory conditions [14,34,44].

Conclusion. In light of growing evidence that mesenchymal stem cells can act as effective drug-delivery platforms, our findings suggest that systemically administered MSCs can be used as targeted immunomodulatory vectors that dampen chronic neuroinflammation at the neurovascular interface and restore cognitive function. Importantly, the feasibility of early intravenous administration highlights MSC-based therapy as a clinically practical and minimally invasive approach with the potential to preserve neural circuit integrity and slow disease progression across a range of inflammation-driven neurological disorders.

## 4. Materials and Methods

### 4.1. Animals

Adult male C57BL/6J mice (8–10 weeks old, 22–25 g) were housed in the animal facility of the Scientific Research Centre of Azerbaijan Medical University under standard laboratory conditions in quiet, temperature-controlled rooms (12 h light/dark cycle) with ad libitum access to food and water. All procedures, including intravenous and intraperitoneal injections, behavioral experiments, as well as decapitation of animals, were carried out in accordance with the ethical guidelines of Directive 2010/63/EU for animal experiments and were approved by the Institutional Animal Care Committee of Azerbaijan Medical University.

### 4.2. Experimental Design

Following a two-week acclimatization period, eight-week-old male mice were randomly divided into three groups: naïve control, LPS + vehicle (Veh), and LPS + MSC treatment (*n* = 15 per group). To induce chronic neuroinflammation, animals in the LPS + Veh and LPS + MSC groups received intraperitoneal injections of lipopolysaccharide (LPS; *Escherichia coli* serotype O111:B4, Sigma–Aldrich, St. Louis, MO, USA) at a dose of 0.75 mg/kg per day for seven consecutive days (days 1–7), in line with previously established protocols [5]. Mice were kept warm after each administration to prevent stress-related complications. Control mice received equal volumes of sterile phosphate-buffered saline (PBS). To verify the inflammatory state at the time of intervention, a separate cohort of animals (*n* = 6) was assessed on day 8 for serum and brain cytokine levels immediately prior to mesenchymal stem cell (MSC) administration. On day 8, following the final LPS injection, animals were randomly allocated to receive a single intravenous treatment with either vehicle or human umbilical cord-derived mesenchymal stem cells (UC-MSCs). A total of 1 × 10^6^ second-passage MSCs were resuspended in 100 µL sterile PBS and administered via the lateral tail vein. Cognitive and memory performance were assessed 14 days after MSC administration (days 22–23 after the first LPS injection) using the open-field, Y-maze spontaneous alternation, novel object recognition, and passive avoidance tests. Animals were euthanized on day 25, and brain tissues were collected for immunohistochemical and biochemical analyses.

### 4.3. Mesenchymal Stem Cell Isolation, Culture and Characterization

Fresh human umbilical cords were obtained from full-term deliveries (39–40 weeks of gestation) with informed donor consent for research use and processed within 12 h of delivery. After thorough rinsing in sterile Dulbecco’s phosphate-buffered saline (PBS) supplemented with antibiotics (VivaCell Biosciences, Freiburg, Germany), Wharton’s jelly was dissected away from the vessels, minced into ~1 mm^3^ fragments, and incubated in α-MEM (VivaCell Biosciences) containing 1 mg/mL collagenase type I and 0.25 mg/mL hyaluronidase at 37 °C for 60 min with gentle agitation. The tissue digest was filtered through a 70 µm strainer, centrifuged, and the resulting cell pellet was resuspended in α-MEM supplemented with 10% fetal bovine serum (FBS), 100 U/mL penicillin, and 100 µg/mL streptomycin (Biological Industries, Cromwell, CT, USA). Cells were seeded into tissue-culture flasks and maintained at 37 °C in 5% CO_2_. Non-adherent cells were removed after 48 h, and adherent fibroblast-like cells were allowed to expand. When cultures reached 80–90% confluence, cells were detached with 0.25% trypsin–EDTA and subcultured at a ratio of 1:3.

For all experiments and injections, early-passage human umbilical-cord MSCs (P2–P3) were used. Immediately before administration, cell suspensions were assessed by Trypan Blue exclusion, and only preparations with a viability of ≥90% were released for use. The target dose of 1 × 10^6^ viable cells in 100 µL PBS was injected via the lateral tail vein. No cell clumping was observed, and all preparations were visually confirmed to be homogeneous before infusion. Third-passage cells were used for all characterization assays.

### 4.4. Flow Cytometry Characterization of Surface Markers in MSCs

Passage 3 UC-MSCs were harvested, washed twice in PBS containing 2% FBS (FACS buffer), and resuspended at 1 × 10^6^ cells/mL. Cells were incubated for 30 min at 4 °C with fluorochrome-conjugated monoclonal antibodies against canonical MSC markers (CD73, CD90, CD105, CD29, CD44, CD146) and negative lineage markers (CD34, CD45, CD11b, HLA-DR). Isotype-matched IgG antibodies served as negative controls. After staining, cells were washed, resuspended in FACS buffer containing 1 µg/mL propidium iodide to exclude dead cells, and analyzed on a BD FACSAria flow cytometer using FlowJo software (version 11.0.2). MSCs were considered positive for a marker when >95% of the population showed fluorescence above the isotype control. As expected, cells were strongly positive for CD73, CD90, CD105, CD29, CD44, and CD146, and negative for CD34, CD45, CD11b, and HLA-DR.

### 4.5. Cytokine Profiling

To assess cytokine secretion, UC-MSCs were seeded at 2 × 10^5^ cells/well in six-well plates and cultured for 48 h in serum-free α-MEM. Supernatants were collected, centrifuged at 1500× *g* for 10 min to remove debris, and stored at –80 °C. Concentrations of IL-5, IL-6, and IL-8 were quantified using a BD Cytometric Bead Array (BD Biosciences, Franklin Lakes, NJ, USA) according to the manufacturer’s instructions. Cytokine levels were below the detection limit, indicating that MSCs remained in a quiescent, non-activated state consistent with a low-immunogenic, anti-inflammatory phenotype.

### 4.6. Assessment of Systemic and Central Inflammatory Status at the Time of MSC Administration

To verify the magnitude of systemic inflammation and neuroinflammation at the time of mesenchymal stem cell (MSC) treatment, a separate subset of mice from the naïve control and LPS-treated groups (*n* = 6 per group) was sacrificed 24 h after the final LPS injection. Blood and brain tissues were collected for biochemical analyses. Whole blood was allowed to clot, centrifuged, and serum samples were stored at −80 °C until analysis. Brains were rapidly removed, snap-frozen, and stored at −80 °C; hippocampi were subsequently microdissected for cytokine measurements.

For tissue processing, hippocampal samples were homogenized on ice by brief sonication (15–20 s) in tissue extraction buffer (Thermo Fisher Scientific, Waltham, MA, USA) supplemented with protease and phosphatase inhibitor cocktails (Sigma-Aldrich, St. Louis, MO, USA). Homogenates were centrifuged at 15,000× *g* for 10 min at 4 °C, and the resulting supernatants were aliquoted and stored at −80 °C until use. Levels of the pro-inflammatory cytokines TNF-α, IL-1β, and IL-6 were quantified to confirm the inflammatory status at the initiation of MSC therapy.

#### 4.6.1. Behavioral Assessments

Behavioral assessments were conducted on Days 22 and 23 following the initial LPS injection, corresponding to Days 14–15 post-MSC administration. This timeline was selected to ensure sufficient duration for mesenchymal stem cell (MSC) homing, paracrine signaling, and neurotrophic support, as well as to allow early detection of functional improvements and cognitive recovery before tissue harvesting on Day 25. The selected behavioral tasks targeted key domains affected by neuroinflammation, including spatial working memory, recognition memory, and aversive learning. In all experiments, the experimenters were blinded to treatment groups during scoring.

#### 4.6.2. Assessment of Spontaneous Locomotor Activity and Anxiety-like Behavior

First, animals in all groups were tested in the open-field test. Before testing, they were habituated to the behavioral suite for at least 30 min to minimize stress. Each mouse was then placed individually into the center of a white acrylic arena measuring 40 × 40 × 40 cm (length × width × height). The arena was illuminated at a uniform intensity of approximately 50 lux, and a constant background of white noise was played to mask external sounds. Mice were allowed to explore freely for 5 min while a ceiling-mounted infrared camera recorded their trajectories. Video data were analyzed using AnyMaze software (version 7.44) to determine total distance travelled, average velocity, and the amount of time spent in the central versus peripheral zones, which together provide indices of locomotor activity and anxiety. The apparatus was thoroughly cleaned between subjects to remove olfactory cues.

#### 4.6.3. Assessment of Spatial Working Memory

The Y-maze test was performed to evaluate spontaneous alternation behavior, a validated measure of spatial working memory [17,18]. The apparatus consisted of three arms (40 cm long, 8 cm wide, 15 cm high) arranged at 120° angles to form a Y-shaped maze. Testing occurred in a quiet room to minimize external interference. On Day 22, each mouse was placed in the central triangular zone, oriented away from a random arm, and allowed to explore freely for 7 min. Arm entries and sequence were recorded via an overhead camera. The maze was cleaned with 70% ethanol between trials. Two outcomes were assessed: (1) the total number of arm entries, reflecting general locomotion, and (2) the percentage of spontaneous alternation, reflecting working memory. A spontaneous alternation was defined as three consecutive entries into three different arms (e.g., A → B → C). The spontaneous alternation percentage was calculated using the formula: (Number of alternation triads/[Total arm entries − 2]) × 100%. Reduced alternation rates in LPS-treated mice indicate working memory impairment, while normalization in MSC-treated animals suggests therapeutic efficacy.

#### 4.6.4. Assessment of Recognition Memory: Novel Object Recognition (NOR) Test

The Novel Object Recognition test was used to evaluate recognition memory, which is particularly sensitive to hippocampal dysfunction. The test was conducted in a 40 × 40 × 40 cm Plexiglas open field placed in a dim, quiet room. On Day 20, mice were habituated to the empty arena for 10 min. On Day 21, the familiarization phase was conducted by placing two identical objects symmetrically in the arena and allowing the mouse to explore for 10 min. To evaluate the long-term memory retention after a 24-h delay on Day 22, one familiar object was replaced with a novel object, and exploration was recorded for 10 min.

During both the familiarization and test phases of the Novel Object Recognition test, the frequency of exploration directed toward each object was quantified to ensure equal opportunity for interaction. The percentage of sniffing behavior for either the novel or familiar object was calculated using the following formula:

Sniffing frequency (%) = (number of sniffing instances toward novel or familiar object/total sniffing events toward both objects) × 100, whereas the recognition index was calculated as the number of sniffing instances toward novel/total sniffing events toward both objects. The Discrimination Index was calculated as: (Tnovel − Tfamiliar)/(Tnovel + Tfamiliar). A positive DI indicated intact recognition memory. Total exploration time was also measured to rule out locomotor confounds.

Exploratory behavior was operationally defined as the animal orienting its nose within 2 cm of the object or making direct nasal contact. Behaviors such as turning away, climbing over, or sitting on the object were excluded from exploration metrics. The duration of object exploration during the test session was automatically recorded and analyzed using the AnyMaze software video tracking system. Object positions were counterbalanced across groups, and all items were cleaned with 70% ethanol between trials.

#### 4.6.5. Assessment of Associative Learning and Long-Term Memory: Passive Avoidance Test

The Passive Avoidance Test (PAT) was conducted to evaluate associative learning and long-term memory, cognitive functions that depend primarily on the hippocampus and amygdala. Testing took place between Days 22–24 post-LPS using a standard step-through shuttle box consisting of an illuminated and a dark compartment (20 × 30 × 20 cm), separated by a guillotine door (6 × 8 cm). The dark chamber was equipped with a stainless-steel grid floor made of 3.175-mm rods spaced 8 mm apart to deliver the foot shock.

On Day 22 (habituation), each mouse was placed in the illuminated compartment with the door open for 5 min, allowing free exploration of both chambers without receiving a shock. This step familiarized the animals with the apparatus and minimized novelty-induced anxiety. On Day 23 (training trial), mice were placed individually in the illuminated chamber facing away from the dark compartment. After a 10 s adaptation period, the guillotine door was lifted. When the mouse fully entered the dark compartment with all four paws, a mild foot shock (0.3 mA, 3-s duration) was automatically delivered. Animals displaying an initial step-through latency exceeding 60 s were excluded from analysis to avoid confounding effects of extreme anxiety or hypoactivity. After shock delivery, mice were immediately removed from the apparatus and returned to their home cages. On Day 24 (retention test), mice were again placed in the illuminated compartment, and the latency to enter the dark side—defined as the step-through latency (STL)—was recorded, with a maximum cutoff of 300 s. No shock was administered during this trial. Higher STL values were interpreted as better memory retention and reduced cognitive impairment. Error counts (re-entries into the dark compartment) were also recorded as an additional measure of retention failure when applicable. All apparatus surfaces were cleaned thoroughly with 70% ethanol between trials to eliminate odor cues. Animals were tested in a randomized order, and experimenters were fully blinded to group assignments to prevent bias.

#### 4.6.6. Tissue Collection

At Day 25, animals were deeply anesthetized via intraperitoneal injection using ketamine hydrochloride (10 mg/mL, Richter Gedeon, Budapest, Hungary) and xylazine (1.17 mg/mL, Alfasan, Woerden, The Netherlands). Once deep anesthesia was confirmed, transcardial perfusion was performed using a 0.9% sodium chloride solution containing 5 mM EDTA over a period of five minutes. This was followed by a perfusion with 4% paraformaldehyde (PFA) in phosphate-buffered saline (PBS) to achieve tissue fixation.

The brains were carefully dissected and post-fixed in 4% PFA overnight at 4 °C. Subsequently, tissues were rinsed in PBS and cryoprotected in 30% sucrose for 48 to 72 h at 4 °C. Following cryoprotection, samples were embedded in optimum cutting temperature (OCT) compound (Tissue-Tek, Sakura Finetek, Alphen aan den Rijn, The Netherlands). Cryosectioning was performed using a Leica CM1850 cryostat (Leica Biosystems, Nußloch, Germany) equipped with Feather A35 disposable blades (Feather, Tokyo, Japan). Brain tissues were cut into 20 μm-thick coronal sections. All cryosections were stored at −80 °C until required for further histological and immunohistochemical analysis.

Additionally, brain tissues were processed for paraffin embedding. After transcardial perfusion with 4% paraformaldehyde (PFA) in PBS, and overnight post-fixation at 4 °C. The fixed brains were dehydrated using a graded ethanol series, followed by clearing in xylene twice for 30 min each and immersion in molten paraffin wax at 60 °C for 2 h (Bio Optica, Milano, Italy). The brains were then embedded in paraffin using stainless steel molds and allowed to solidify at room temperature. Serial coronal sections of 5 to 7 μm thickness were obtained using a rotary microtome HM325 (Thermo Scientific, Waltham, MA, USA) and mounted on Superfrost Plus microscope slides. The mounted slides were dried overnight at 37 °C before proceeding with staining protocols.

#### 4.6.7. Histological and Immunohistochemical Studies

For hematoxylin and eosin (H&E) staining, paraffin sections were deparaffinized in xylene and rehydrated through a descending ethanol series into distilled water. Hematoxylin staining was performed for six minutes, followed by a ten-minute tap water rinse to allow for nuclear blueing. Eosin staining was performed for two minutes using 1% Eosin Y solution. After staining, the slides were dehydrated through ascending ethanol concentrations, cleared in xylene, and mounted using DPX mounting medium (BDH or Merck, Darmstadt, Germany).

Obtained cryosections and paraffin sections were used for immunohistochemical and immunofluorescence analysis as described previously. To block endogenous peroxidase activity, sections were incubated for 10 min in 0.3% hydrogen peroxide prepared in methanol. Sections were then washed in Tris-buffered saline (TBS; 50 mM, pH 7.4) containing 0.01% Triton X-100. Non-specific binding was minimized by blocking for 1 h in either 5% normal goat serum (Bio-Rad, Hercules, CA, USA) in PBS (for paraffin sections) or TBS containing 10% fetal bovine serum, 3% bovine serum albumin, and Triton X-100 (1% for light microscopy) for free-floating sections. Primary antibody incubations were performed overnight at 4 °C in the corresponding blocking buffer. The following primary antibodies were used: goat anti-IBA-1 (1:1000, Abcam, Cambridge, MA, USA), mouse anti-CD45 (1:100, Abcam), and rabbit anti-GFAP (1:3000, Agilent Tech, Carpinteria, CA, USA).

After rinsing, sections were incubated with the appropriate biotinylated secondary antibodies (Vector Laboratories, Newark, CA, USA; 1:200) for 1–1.5 h at room temperature, followed by signal amplification with the VECTASTAIN^®^ ABC reagent (Vector Laboratories; 1:100). Immunoreactivity was visualized using 3,3′-diaminobenzidine (DAB; 0.05%) with 0.015% hydrogen peroxide in Tris buffer (0.05 M, pH 8.0) for 5 min. Finally, sections were counterstained with hematoxylin, dehydrated, cleared, and coverslipped for light microscopy. Microscopic evaluation was performed using a Leica DM500 light microscope, and images were captured with a Canon digital camera (Canon Inc., Tokyo, Japan).

Ionized calcium-binding adaptor molecule 1 (IBA1)–immunoreactive microglia and glial fibrillary acidic protein (GFAP)–immunopositive astrocytes are visualized by immunohistochemistry in every 20th coronal section spanning the entire rostrocaudal extent of the hippocampus. We quantified the densities of IBA-1^+^ microglia per unit volume (per 0.1 mm^3^) using three serial sections through the mid-region of the hippocampus in each group of animals (*n* = 6 animals per group) using a StereoInvestigator system comprising a color digital video camera interfaced with a Zeiss Primo Star or Leica DM500 light microscope. The area fractions occupied by GFAP^+^ immunoreactive astrocytes and CD45^+^ puncta in the hippocampal CA1 subfield and the prefrontal cortex area (for CD45^+^ puncta) were evaluated using ImageJ software (version 1.54), employing color deconvolution and automated thresholding. From each region, 5 sections per animal were analyzed (*n* = 6 animals per group).

#### 4.6.8. Dual Immunofluorescence Staining

To evaluate the percentage of microglia co-expressing IBA-1 and CD68, a dual immunofluorescence staining protocol was applied. Briefly, sections were first incubated with a cocktail of goat anti-IBA-1 (1:1000, Abcam, Cambridge, UK) and rat anti-ED-1/CD68 (1:500, Bio-Rad, Hercules, CA, USA) primary antibodies, followed by incubation with donkey anti-goat IgG conjugated to Alexa Fluor 488 (1:200, Invitrogen, Waltham, MA, USA) and donkey anti-rat IgG conjugated to Alexa Fluor 594 (1:200, Invitrogen, USA). Fluorescence signals were visualized using a confocal microscope, and colocalization of IBA-1 and CD68 signals was quantified as the ratio of IBA-1^+^ and CD68^+^ area to total IBA-1^+^ area. The percentages of microglia expressing both IBA-1 and CD68 were quantified through Z-section analysis in a Carl Zeiss LSM900 Confocal Microscope (Zeiss, Jena, Germany).

#### 4.6.9. Biochemical Analysis

A separate cohort of animals (*n* = 6 per group) is deeply anesthetized on the day of sacrifice. Brains are rapidly removed, and the hippocampi are carefully microdissected, snap frozen in liquid nitrogen, and stored at –80 °C until biochemical analysis. For tissue preparation, frozen hippocampi are homogenized on ice in Tissue Extraction Buffer (Invitrogen, USA) supplemented with a protease inhibitor cocktail (Sigma-Aldrich/Merck, Darmstadt, Germany) using a sonic dismembrator (Sigma-Aldrich; 30 s, 1:100 dilution). The homogenates are centrifuged at 15,000× *g* for 10 min at 4 °C, and the supernatants are aliquoted and stored at –80 °C. Protein concentrations are quantified using the Pierce™ BCA Protein Assay Kit (Thermo Fisher Scientific, USA), and equal amounts of total protein from each sample are used for cytokine determination.

Levels of pro-inflammatory cytokines tumor necrosis factor-α (TNF-α), interleukin-1β (IL-1β), and interleukin-18 (IL-18), as well as the anti-inflammatory cytokine interleukin-10 (IL-10), were quantified in hippocampal tissue homogenates using enzyme-linked immunosorbent assay (ELISA) kits, according to the manufacturers’ instructions. ELISA kits for TNF-α, IL-1β, IL-18, and IL-10 were obtained from Sigma-Aldrich/Merck (Germany). Absorbance was measured using a microplate reader, and cytokine concentrations were calculated from standard curves and normalized to total protein content.

#### 4.6.10. Statistical Analysis

Data are presented as mean ± SEM. Statistical analyses were performed using GraphPad Prism 10 and SPSS 26. Normality was assessed using the Shapiro–Wilk test. Normally distributed data were analyzed by one-way ANOVA with Tukey’s post hoc test, while non-parametric data were analyzed using the Kruskal–Wallis test with Dunn’s correction. Repeated-measures ANOVA was applied where appropriate, and two-group comparisons were performed using unpaired two-tailed Student’s *t*-tests. Statistical significance was set at *p* < 0.05.

## Data Availability

The original contributions presented in this study are included in the article/Supplementary Material. Further inquiries can be directed to the corresponding author.

## Supplementary Materials

The following supporting information can be downloaded at: https://www.mdpi.com/article/10.3390/ijms27031182/s1.

## Abbreviations

The following abbreviations are used in this manuscript:

AD	Alzheimer’s disease
Ang-1	Angiopoietin-1
ANOVA	Analysis of variance
BBB	Blood–brain barrier
BDNF	Brain-Derived Neurotrophic Factor
CNS	Central nervous system
DI	Discrimination index
ELISA	Enzyme-linked immunosorbent assay
GFAP	Glial fibrillary acidic protein
H&E	Hematoxylin and eosin
Iba1	Ionized calcium-binding adapter molecule 1
IL	Interleukin
i.p.	Intraperitoneal
i.v.	Intravenous
LPS	Lipopolysaccharide
MAPK	Mitogen-activated protein kinase
MSC	Mesenchymal stem cell
NGF	Nerve Growth Factor
NORT	Novel object recognition test
OFT	Open-field test
PAT	Passive avoidance test
PBS	Phosphate-buffered saline
PFA	Paraformaldehyde
SA	Spontaneous alternation
TLR4	Toll-like receptor 4
TNF-α	Tumor necrosis factor alpha
UC-MSC	Umbilical cord-derived mesenchymal stem cell
VEGF	Vascular Endothelial Growth Factor
