# Supplementary material for: Mesenchymal Stem Cell Therapy Modulates Peripheral–Central Immune Interactions and Attenuates Neuroinflammation-Driven Cognitive Dysfunction
Source: Int J Mol Sci. 2026 Jan 24;27(3):1182. doi: 10.3390/ijms27031182 (PMC12897210; doi:10.3390/ijms27031182)
Supplement: Supplementary file 1 [file ijms-27-01182-s001.zip › ijms-4107753-supplementary.pdf]

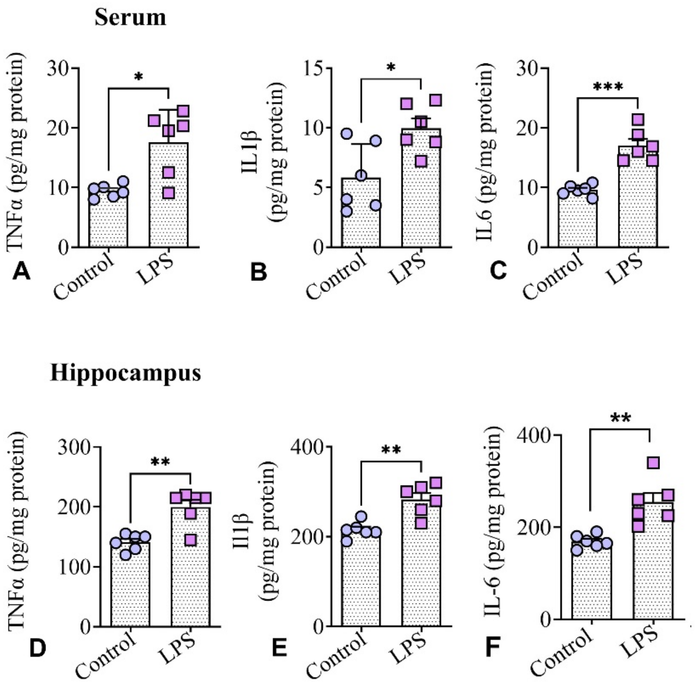

Supplementary Figure S1. Administration of lipopolysaccharide elicited robust systemic and peripheral inflammatory activation before MSC intervention. Bar graphs A–F illustrate serum (A–C) and hippocampal (D–F) concentrations of the pro-inflammatory cytokines TNF-α (A, D), IL-1β (B, E), and IL-6 (C, F). *, $p < 0.05$; **, $p < 0.01$; ***, $p < 0.001$.

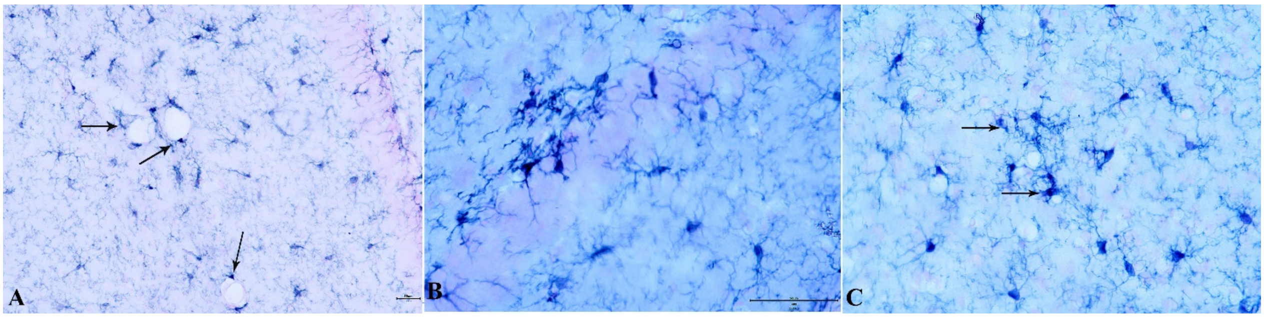

Supplementary Figure S2. Perivascular clustering of microglia in hippocampal and prefrontal regions following chronic LPS exposure. Representative IBA1 immunohistochemical images from vehicle-treated LPS (LPS-Veh) mice showing microglial clustering patterns. Panels **A** and **C** illustrate IBA1^{+} microglia aggregated in proximity to blood vessels (arrows), consistent with perivascular accumulation, while panel **B** shows clustered microglia within regions enriched in neuronal cell bodies. Scale bars = 50 µm.
